# Comparison of Clinical, Radiological and Laboratory Parameters Between Elderly and Young Patient With Newly Diagnosed Smear Positive Pulmonary Tuberculosis: A Hospital-Based Cross Sectional Study

**DOI:** 10.7759/cureus.8319

**Published:** 2020-05-27

**Authors:** Manju Rajaram, Archana Malik, Madhusmita Mohanty Mohapatra, Mathavaswami Vijayageetha, Vemuri Mahesh Babu, Soundara Vally, Vinod Kumar Saka

**Affiliations:** 1 Pulmonary Medicine, Jawaharlal Institute of Postgraduate Medical Education and Research, Puducherry, IND; 2 Internal Medicine, Jawaharlal Institute of Postgraduate Medical Education and Research, Puducherry, IND; 3 Preventive Medicine, Jawaharlal Institute of Postgraduate Medical Education and Research, Puducherry, IND

**Keywords:** tuberculosis, rntcp, elderly vs younger age group, sputum positive, chest x-ray

## Abstract

Introduction

To compare clinical, radiological and haematological manifestations among newly diagnosed smear positive tuberculosis patients between Group I (Elderly >60 yrs) and Group II (Younger age between 13 and 60 years).

Methodology

This was a hospital-based cross-sectional study conducted at the out-patient department of pulmonary medicine, between March 2014 and December 2017. There were 61 patients in Group I (Elderly > 60 yrs) and 110 patients in Group II (Younger age between 13 and 60 years). Continuous variables were compared using student’s t-test and Mann-Whitney test. Chi square test and Fischer test was used for analysing categorical variables. All statistics were two-tailed, and a p-value of 0.05 was considered to be statistically significant.

Results

The mean age for Group I (Elderly >60 yrs) was 65 ± 2 years and for the Group II (Younger age between 13 and 60 years) was 40 ± 1 years. There was a statistically significant association of cavitation with infiltrates (p = 0.007) in younger age group. Bilateral multiple zone (48, 64.86%) involvements were commonly observed in both the age groups. There was no significant difference between two groups with regard to haematological and clinical parameters.

Conclusion

We did not find any difference in the presentation of tuberculosis in both the groups. Radiologically, there was more of cavitating lesion in younger age group. So, they should be isolated and followed up at regular intervals.

## Introduction

Tuberculosis is still a major threat to our society even after Revised National Tuberculosis Control Program (RNTCP) was started in India in 1997, and the whole nation was covered in March 2006. India contributes to one-fourth of the global burden of tuberculosis. Annually, approximately 220,000 people die due to tuberculosis, that is two deaths every five minutes [1]. India is considered “an aging nation” with 7.7% of its population older than 60 years of age. It is estimated that tuberculosis in the elderly comprises up to 14% of all cases [2]. The prognosis of tuberculosis in the geriatric population is grave due to drug-related adverse events, increased comorbidity and a higher rate of poverty than younger adults.

The presentation of tuberculosis varies across different age groups. As compared to a younger age group, atypical symptoms like dyspnea, weight loss, and malaise are more common in the elderly [3]. Tuberculosis mortality was higher in an elderly age group of patients in compared to a younger age group of patients [4].

Although the literature on comparing the manifestation of tuberculosis in both the young and elderly is available, the results are discordant. There is also a scarcity of Indian literature on the comparison of different manifestations of tuberculosis between an elderly and young population. Hence, this study was conducted to compare clinical, radiological, and hematological manifestations between elderly (>60 years old) and young (13 to 60 years old) newly diagnosed smear-positive tuberculosis patients to facilitate early detection of cases and prompt management.

## Materials and methods

Study setting

This study was conducted at the pulmonary medicine out-patient department of a tertiary care hospital located in Puducherry on the southeastern coast of India.

Study design

This was a hospital-based cross-sectional study conducted from March 2014 to December 2017. The sample size was estimated using a statistical formula for comparing proportions and an expected difference in manifestation between the groups of 20%. The minimal sample size required for the study was estimated as 79 in each group providing a 5% level of significance and 80% power. To account for a non-response rate of 10%, 87 participants were taken in each group [5]. However, within the study period, only a total of 171 patients with smear-positive pulmonary tuberculosis were included. There were 61 patients in Group I (>60 years old) and 110 patients in Group II (age 13 to 60 years old). Patients infected with human immunodeficiency virus, patients with a known case of any malignancy, patients with a history of chronic obstructive pulmonary disease or bronchial asthma were excluded.

Written informed consent was obtained from each participant. For minor participants, assent was determined, and consent was provided by their parents. The confidentiality of personal identity information was maintained throughout this study procedure.

Study procedure

Information regarding socio-demographic details, presenting symptoms, and clinical examination were collected. Chest X-ray and biochemical tests such as complete hemogram, liver function test (LFT), random blood sugar, and renal function test (RFT) were performed. Chest X-rays of all patients were reviewed for a predominant pattern of opacity like fibrosis, cavity, infiltrate, miliary opacity, the zone of involvement, and extent of pulmonary lesions. Based on the extent of the lesion, chest X-rays were classified into three types: I) minimal lesion, II) moderately advanced lesion, and III) far advanced lesion [6].

Operational definitions

Minimal lesion was defined as a single lesion in the absence of cavity. The total extent of the lesion, regardless of the distribution, shall not exceed the equivalent of lung tissue which lies above the second costosternal junction and spine of fourth or body of fifth thoracic vertebrae on one side [6].

Moderately advanced was defined as one or both lungs may be involved, but the extent of the lesion shall not exceed the following limits:

1. A slightly disseminated lesion that may not exceed more than the volume of one lung or the equivalent of this in both lungs.

2. A dense confluent lesion that may not exceed more than equal to one-third of the lung or any gradation within the above limits.

3. The total diameter of the cavity if present should not be more than 4 cm [6].

Far advanced lesion was defined as a lesion more advanced than the moderately advanced type [6].

A hemoglobin level of less than 13 g/dl for men and less than 12 g/dl for women was considered as low hemoglobin.

This study was approved by the Institute Ethics committee.

Statistical analysis

Data were entered in Epidata version 4.1. Descriptive analysis was performed using STATA version 14.1 (StataCorp LLC, College Station, TX). After assessing for normality, continuous data were summarized as either mean or standard deviation or median and interquartile range. Categorical data were summarized as proportions. Based on the normality distribution, continuous variables were compared using Student’s t-test and Mann-Whitney U test. A chi-square test and Fischer’s test were used for analyzing categorical variables. Univariate analysis was used for calculating unadjusted odds ratios. All statistics were two-tailed, and p < 0.05 was considered statistically significant.

## Results

The demographic parameters of the patients in two groups have been shown in Table 1. Majority of the patients in our study were males and the mean age for group I (Elderly >60 yrs) was 65 ± 2 years and for the group II (Younger age between 13 and 60 years) was 40 ± 1 years. Among smokers, 61 (60%) patients were in younger age group and 40 (40%) were in the elderly group and there was no statistically significant association observed between the groups (p > 0.05). Younger age group patients (Group II) had 1.5 times higher chance of being a non-smoker (odds ratio = 1.5). Diabetes mellitus was the most common co-morbidity among both groups. There was no statistically significant association of any of the co-morbidities among the two groups.

**Table 1:** Baseline characteristics of smear positive pulmonary tuberculosis patients between younger and elderly age group of patients attending a tertiary care facility, Puducherry, South India. Group I (Elderly > 60 yrs) and Group II (Younger age between 13 and 60 years) CKD: Chronic kidney diseases; CLD: Chronic liver diseases.

	Group-I n (%)	Group-II n (%)	p-value	Odds ratio
Male	57 (38.5)	91 (61.5)	0.04	
Smoker	40 (40)	61 (60)	0.199	1.53
Diabetes	14 (40)	21 (60)	0.275	0.60
Hypertension	2 (100)	0		
CKD	4 (57.13)	3 (42.16)	0.064	0.30
CLD	1 (50)	1 (50)	0.024	0.40

Table 2 shows symptom wise comparison of patients in both groups. Among the elderly, 60 (35.97%) and 107 (64.07%) among younger adults had cough. Fever was more commonly found in younger age group in 59 patients (60.82%) as compared to elderly 38 patients (39.18%). There was no statistically significant association of symptoms between the two groups. Younger age group patients had a statistically significant association between auscultatory findings like bronchial breath sound and decreased intensity as compared to elderly age group (p-value 0.038 and 0.01, respectively) which was shown in Table 3.

**Table 2:** Presenting clinical symptoms of smear positive pulmonary tuberculosis patients between younger and elderly age group of patients attending a tertiary care facility, Puducherry, South India. Group I (Elderly > 60 years) and Group II (Younger age between 13 and 60 years)

	Group I	Group II	p-value
Symptoms			
Cough	60 (35.97)	107 (64.07)	0.852
Fever	38 (39.18)	59 (60.82)	0.33
Hemoptysis	10 (45.45)	12 (54.55)	0.32
Constitutional symptoms	24 (33.80)	47 (66.20)	0.607
Breathlessness	9 (52.94)	8 (47.06)	0.126

**Table 3:** Auscultatory findings of smear positive pulmonary tuberculosis patients between younger and elderly age group of patients attending a tertiary care facility, Puducherry, South India. Group I (Elderly > 60 years) and Group II (Younger age between 13 and 60 years)

Auscultatory findings	Group I	Group II	p-value	Odds ratio
Normal vesicular	6 (18.75)	26 (81.25)	0.68	
Bronchial	27 (40.3)	40 (59.7)	0.038	0.34
Decreased intensity	5 (71.43)	2 (28.57)	0.01	0.092
Crepitations	23 (35.38)	42 (64.62)	0.09	0.421

The most common X-ray finding in younger age group was presence of infiltrates in chest X-ray (48, 65.76%) whereas consolidation was the most common X-ray finding among the elderly (14, 56%). There was a statistically significant association of cavitation with infiltrates (p = 0.007) in younger age group. Younger adults had 2.25 times higher chance of minimal radiological involvement and there was no statistically significant association with regard to extent of involvement between the two groups. Upper zone (17, 54.84%) and bilateral multiple zone (48, 64.86%) involvement was commonly observed in X-ray finding in younger adults. There was no significant association in involvement of zone between two groups (Tables 4-6).

**Table 4:** Comparison of type of opacities in radiology between elderly and younger age group of patients attending a tertiary care facility in Puducherry, South India.

Type of opacities	Group I n (%)	Group II n (%)	p-value	Odds ratio
Consolidation	14 (56)	11 (44)	0.549	0.785
Cavitation	9 (45)	11 (55)	0.464	1.55
Infiltrates	25 (34.25)	48 (65.76)	0.05	2.44
Miliary shadows	1 (100)	0 (0)	0	0
Cavitation with infiltrates	11 (23.4)	36 (76.6)	0.007	4.165

**Table 5:** Comparison of extent of involvement of lung by radiology between elderly and younger age group of patients attending a tertiary care facility in Puducherry, South India.

Extent of involvement	Group I n (%)	Group II n (%)	p-value	Odds ratio (95% confidence interval)
Minimal	12 (30.77)	27 (69.23)	0.524	2.25
Moderate	29 (36.71)	50 (63.29)	0.436	0.76
Advanced	19 (38.78)	30 (61.22)	0.81	0.71
Normal	1 (25)	3 (75)	0.019	1.33

**Table 6:** Comparison of site of involvement by radiology between elderly and younger age group of patients attending a tertiary care facility in Puducherry, South India.

Site of involvement	Group I n (%)	Group II n (%)	p-value
Upper	14 (45.16)	17 (54.84)	0.699
Lower	0 (0)	1 (100)	0
Middle	1 (25)	3 (75)	0.45
Unilateral multiple	20 (32.79)	41 (67.21)	0.24
Bilateral multiple	26 (35.14)	48 (64.86)	0.33

Younger adult had 2.19 times higher chance of having a positive scanty smear status. There was no statistically significant association of smear status among the groups. There is no significant difference between two groups with regard to hematological parameters (Table 7).

**Table 7:** Comparison of laboratory parameters between elderly and younger age group of patients attending a tertiary care facility, Puducherry, South India. Group I (Elderly > 60 years) and Group II (Younger age between 13 and 60 years)

Sputum status	Group I n (%)	Group II n (%)	p-value	Odds ratio
Scanty	3 (23.08)	10 (76.92)	0.067	2.19
1+	15 (46.88)	17 (53.13)	0.149	0.35
2+	18 (47.37)	20 (52.63)	0.135	0.35
3+	25 (29.4)	63 (71.6)	0.689	0.75
	Group I n (%)	Group II n (%)	p-value	
Low Hb%	29 (29.59)	69 (70.4)	0.26	
Normal Hb%	32 (43.84)	41 (56.16)		
Abnormal liver function	3 (75)	1 (25)	0.45	
Normal liver function	58 (35.67)	109 (65.27)		

## Discussion

The majority of the patients in our study were men, similar to the studies done by Bhushan et al. and Gupta et al. [7,8]. The male predominance among both the groups is probably because of the higher level of outdoor and social activities of men as compared to women. Smoking has no association with any of the groups in our study population; this is concordant with Gupta et al. and Pérez-Guzmán et al. [8,9]. Diabetes mellitus was the most common co-morbidity noticed in our study in agreement with Wang et al. [7,10]. Patients with diabetes mellitus are more susceptible to pulmonary tuberculosis due to neutrophilic dysfunction and impaired cytokine formation.

Cough was the most commonly observed symptom in our study. There was no significant association between any of the symptoms between the two groups. As per a study by Lee et al., there was a significant association of hemoptysis and breathlessness in the younger and elderly age groups, respectively [11]. Studies from Patiala, India as well as from Egypt demonstrated a significant association of fever, hemoptysis, and weight loss in younger patient, and dyspnea and chest pain in the older age group [12,13]. Potential explanations for this disparity may be the variation in patient characteristics, smaller sample size, and single-center data. Other potential explanations could be due to a difficulty in expressing the symptoms in the elderly and the presence of co-morbidities that may mask the symptoms of tuberculosis.

Infiltrates were found to be the most common radiological finding in both younger (35.25%) and elderly (65.76%) patients. Cavitation with infiltration was significantly associated with the younger age group in our study, similar to results from Cantalice Filho et al. [14]. Due to impaired T-cell function and poor immunological status, the elderly are less prone to cavity formation. In contrary to other studies, we found that there was no significant association of involvement of the lower zone in the elderly. However, one study from Brazil found the same result [14]. In elderly, far advanced lesions were commonly observed, probably due to higher immunological susceptibility and delay in the diagnosis of disease.

There was no significant association of hematological parameters in both the elderly and young age groups. Anemia was more commonly associated with the elderly age group [15]. A correlation was not found because, unlike other studies, patients with malignancies and those with human immunodeficiency virus were excluded. Additionally, the sample size was small (n = 171).

Limitations

It was done on patients from an out-patient department and the sample size was small with a single data center.

Strengths

Since the study was done in a tertiary care facility, all patients underwent sputum examination, LFT, RFT and radiological examination.

## Conclusions

Radiologically, there are more cavitating lesions in the younger age group. Therefore, younger patients should be isolated and monitored via follow-up at regular intervals to prevent the spread of infection. We did not find any difference in the presentation of tuberculosis in either of the groups. This may be due to a delay in the diagnosis of tuberculosis and a lack of awareness about the symptoms. Based on our findings, we recommend early screening, an intensified case-finding strategy, and health education for the early diagnosis and management of patients with tuberculosis.
